# Supplementary material for: A systems map of the determinants of child health inequalities in England at the local level
Source: PLoS One. 2021 Feb 12;16(2):e0245577. doi: 10.1371/journal.pone.0245577 (PMC7880458; doi:10.1371/journal.pone.0245577)
Supplement: S1 Appendix — (PDF) [file pone.0245577.s001.pdf]

Appendix 1: Mapping the child health system at the local level online surveys

Details of survey 1

Introductory text:

Thank you for agreeing to take part in an online Delphi Survey. Your responses will help us develop a system map of child health inequalities at the local area level.

The research is led by a team of researchers at the Universities of Sheffield and Bristol and is funded by the NIHR School for Public Health Research (SPHR). It seeks to answer: How can we design better systems to improve child health and reduce inequalities at a local level? It will focus on the development of a system map to support planning for action on child health inequalities.

The research team has been working in partnership with two local areas to develop local system maps of child health inequalities and through this, have developed a 'generic' version that may be useful to other areas in England. The purpose of the Delphi Survey is to work with a wide range of experts in child health, public health, and systems science to refine this map.

The overall aim is to create a child health system map for use at a local level in order to inform opportunities for effective interventions at a systems level to reduce child health inequalities.

The survey will take at least 30 minutes but may take longer. You do not have to answer all the survey questions – you can skip a question and move on to the next.

Consent:

Before we begin, please read the following statements and check the box for each one (you do have to check each box before you can proceed).

- I have read and understood the project information sent by email by the research team
- I understand that taking part in this survey is voluntary
- I understand my personal details such as name, phone number, address and email address etc. will not be revealed to people outside the project.
- I understand my personal details such as name, phone number, address and email address etc. will not be revealed to people outside the project.
- My personal data will be kept by the research team for three years following the end of the study and securely destroyed thereafter.

Thank you.

We would like to collect some details from you so we know who has responded. As we stated, these will be kept confidential within the research team and you will not be identifiable in any publications or other research outputs.

- Please tell us your email address (this is so we can identify which of our invited persons have responded)
- Please tell us your job title:
- Please tell us which sector you are employed in: (please tick one)
    - University or other research institution
    - Local government

- National government or agency
- NHS (including Clinical Commissioning groups)
- Third sector
- Other

- If you selected Other, please specify:
- Please tell us which local authority area your employer is based in:

Some information about systems thinking:

Systems thinking has emerged as a promising approach to public health decision-making, in response to a growing awareness that prevention of health problems needs to reflect the complexity of causes from an individual level through to policy settings. It considers connections among different components, anticipates their interaction, embraces transdisciplinary viewpoints, and requires the active engagement of a wide range of stakeholders.

We provide an example of a system map below. This is a systems map developed in the US, by community leaders, about child health. Each piece of text on the map is a factor that affects child health (we call these nodes).

The arrows reflect how change in one node impacts on one or more other nodes on the map.

(Example map included from Transtria LLC (2014). Evaluation of Healthy Kids, Healthy Communities Cross-Site Report. Accessed at: http://www.transtria.com/hkhc)

We are doing something similar for English local areas. During this survey we will ask you to consider the following key question:

What factors impact child health and wellbeing, and inequalities across these, at the local area level?

Some background:

The research team have been working in two local authority areas to develop area-specific system maps with colleagues from the local authority, NHS, third-sector and others from each area.

We have identified commonalities and differences across these two maps, and used existing research evidence on child health inequalities, in order to develop a generic map that may be used in other local areas in England.

It is this map that we are consulting on in this survey.

Some definitions and guidance:

The focus of the map is: Child health and wellbeing, and inequalities across these, at the local area level.

'Local area': By 'local area' we mean the geographical boundaries of a local authority in England. However components of the system will not be organisationally-bounded to local government but will include other public bodies (e.g. the NHS), third and private sector actors.

Outcomes, children, and inequalities: We would like you to consider all aspects of child health (for example obesity, mental health, oral health, infectious diseases…) for children and young people aged 0-25 years. We also hope to reflect all aspects of inequality, not least socio-economic status, ethnicity, gender, disability, family-type etc

Therefore the boundaries of the system are as follows:

- Children and young people age 0-25
- Children and young people living within the geographical footprint of a local authority
- All aspects of child health
- All dimensions of inequalities

Finally, all the factors on the map must be

- Within influence and/or control of agencies at the local level (e.g. local government, NHS or CCG)
- Capable of change e.g. they must be able to increase or decrease over time

System map of child health inequalities at the local level:

Thank you.

The system map

The link below shows an overview of the system map we have developed after our work in two local areas (link to draft map). You will need to zoom in to see the detail of the map. It may be useful to keep the page with the full map open as you proceed through the survey.

The map represents the factors that influence child health and wellbeing and inequalities in a local authority area. By local area we mean a geographical area bounded by a local authority (though some factors on the map will not be within the remit of local authority governance). It may be helpful to look at the map with a local authority area you are familiar with in mind.

Please take some time to look at it. Factors influencing child health and wellbeing are represented as nodes. It looks busy, but you can see there are different colours on the map. Each colour represents a 'domain'.

The factors on the map have been categorised into six domains using a framework from the literature (Goldfeld, S., Woolcock, G., Katz, I. et al. Neighbourhood Effects Influencing Early Childhood Development: Conceptual Model and Trial Measurement Methodologies from the Kids in Communities Study. Soc Indic Res (2015) 120:197).

We will ask you to comment on each domain separately, so you can consider them in detail.

Nodes are linked if they directly affect each other (our map currently does not show the direction of impact).

Domain Questions:

[Each domain was then shown in turn. The economic domain is shown as an example. The remaining five domains were shown in turn, with the exact same questions.]

Economic domain:

This area of the map represents the economic domain. You can see several nodes e.g. 'area-level deprivation' and connections between the nodes.

Please consider this domain. Remember, each node on the map must be within the influence and/or control of agencies at the local level, and capable of change.

For this domain, please answer the following questions.

- Do you think there are any other important nodes missing from this area of the map? If yes, please add up to 6 nodes in the space below:
- Please comment on your reasons for adding each node mentioned above:
- For each new node you suggested, please tell us how they relate to other nodes in this domain of the map:
- Are there any nodes on this domain of the map you think are incorrect? Please tell us why:

Personal characteristics:

The list below represents a range of personal characteristics and circumstances about children and young people that cannot easily be changed by any intervention. These characteristics are likely to impact on their life experience, engagement with services, and health outcomes (and inequality in outcomes).

- age
- ethnicity
- sexual identity
- gender identity
- cultural/religious background
- English language capability
- low birth weight
- military family
- young/teenage parent
- young carer
- care leaver
- looked-after child
- looked-after child placed outside home local authority
- child at risk
- child/young person experiencing transition
- experience of adverse childhood events (ACEs)
- living with previously unsurvivable illness
- physical disability
- intellectual disability
- complex and/or long-term health condition(s)
- autism spectrum disorder
- SEND
- behavioural difficulties

Please consider these characteristics. Are there any missing, or some that should not be included in this group?

- Please tell us if there are any personal characteristics or circumstances missing:
- Please comment on your reasons for adding each characteristic mentioned above:
- Are there any characteristics in our list that you think should not be there? Please tell us which ones, and why:

- Do you think our systems map is useful in identifying how health inequalities emerge for these groups?

Edge factors:

Our final question about the map is about 'edge factors'.

As a reminder, the focus of the map is the drivers of child health and wellbeing, and inequalities accross these, at the local area level. By 'local area' we mean the geographical boundaries of a local authority in England. The factors on the map should be within influence and/or control of agencies at the local level (e.g. local government, NHS or CCG).

There are some factors identified through the work with two local areas that impact on children and young people's health, but are not within the influence or control of agencies at the local level. These are called 'edge factors'. They include:

- Brexit
- Climate change
- Austerity
- National policy and statutory guidance
- National government formulae
- Statutory inspection regime(s)
- Welfare reform
- Rural/urban geography of area

- Please consider this list of edge factors. Do you think there are any others? If yes, please list below.

The image below shoes how edge factors (the red nodes) might be represented on the system map. They could be linked to nodes in the system that are with in local area control (e.g.Welfare reform is linked to Employment opportunities in the example below); or they could be shown alongside the system map, but not linked to it (e.g. Climate change).

- How best can edge factors be represented on the system map? Please tick one of the following options:
    - As separate nodes on the side of the map, but not linked to the map itself.
    - As separate nodes on the side of the map, with links to nodes on the map
    - They should not be on the map at all

Thank you and contact details:

Thank you, you have completed Round 1 of the Delphi survey.

The research team will collate the feedback from all respondents and refine the map based on these responses.

We will share the next version of the system map with you in a second round of this survey, expected to be in February 2020. You are not obliged to complete this second, final round but we would be very grateful for your feedback on the next version of the map.

In addition, the research team will be conducting qualitative interviews about the utility (or otherwise) of this systems map in addressing child health inequalities in child health at the local level.

If you are interested finding out more about these interviews, please let us know below. Someone from the research team will send full information about taking part to those who have expressed interest.

- Yes, please send me more information about participating in an interview
- No, I do not want further information about the interview

If you have any questions about this study, we would love to hear from you. Please contact the research team by emailing tricia.jessiman@bristol.ac.uk

Thank you.

Details of survey 2

Introductory information:

Thank you for agreeing to take part in this second round of the survey (you may not have taken part last time but are still eligible to complete this survey). Your responses will help us refine a system map of child health inequalities at the local area level.

The research is led by a team of researchers at the Universities of Sheffield and Bristol and is funded by the NIHR School for Public Health Research (SPHR). It seeks to answer: How can we design better systems to improve child health and reduce inequalities at a local level? The overall aim is to create a child health system map for use at a local level in order to inform opportunities for effective interventions at a systems level to reduce child health inequalities.

The research team has been working in partnership with two local areas to develop local system maps of child health inequalities and through this, have developed a 'generic' version that may be useful to other areas in England. This was sent out for consultation in a survey that ended in Jan 2020 and we have refined the map in line with responses received.

The purpose of this second survey is to explain and outline the changes we have made to the map, and ask for final comment from a wide range of experts in child health, public health, and systems science.

The survey will take at least 30 minutes but may take longer. You do not have to answer all the survey questions – you can skip a question and move on to the next.

Consent:

Before we begin, please read the following statements and check the box for each one (you do have to check each box before you can proceed).

- I have read and understood the project information sent by email by the research team
- I understand that taking part in this survey is voluntary
- I understand my personal details such as name, phone number, address and email address etc. will not be revealed to people outside the project.

- I understand my personal details such as name, phone number, address and email address etc. will not be revealed to people outside the project.
- My personal data will be kept by the research team for three years following the end of the study and securely destroyed thereafter.

Thank you.

We would like to collect some details from you so we know who has responded. As we stated, these will be kept confidential within the research team and you will not be identifiable in any publications or other research outputs.

- Please tell us your email address (this is so we can identify which of our invited persons have responded)
- Please tell us your job title:
- Please tell us which sector you are employed in: (please tick one)
    - University or other research institution
    - Local government
    - National government or agency
    - NHS (including Clinical Commissioning groups)
    - Third sector
    - Other
- If you selected Other, please specify:
- Please tell us which local authority area your employer is based in:

Some information about the system map:

Systems thinking has emerged as a promising approach to public health decision-making, in response to a growing awareness that prevention of health problems needs to reflect the complexity of causes from an individual level through to policy settings. It considers connections among different components, anticipates their interaction, embraces transdisciplinary viewpoints, and requires the active engagement of a wide range of stakeholders.The research team have been working in two contrasting local authority areas to develop area-specific system maps with colleagues from the local authority, NHS, third-sector and others from each area.

The key question for the development of the maps has been*: What factors impact child health and wellbeing, and inequalities across these, at the local area level?*

We developed a system map of child health and wellbeing specific to these two local areas. We then identified commonalities and differences across these two maps, and used existing research evidence on child health inequalities, in order to develop a generic map that may be used in other local areas in England.

You can see the map by clicking this link (link to draf map) (you might want to keep it open on a separate browser page while you complete the survey). It looks busy, but you can use the zoom key to see the detail. e consulted on an earlier version of this map during round 1 of this survey. The purpose of this round is to share the feedback and changes we made to the generic map, and ask for further comment.

How the map is structured:

The focus of the map is child health and wellbeing, and inequalities across these, at the local area level.

‘Local area’: By ‘local area’ we mean the geographical boundaries of a local authority in England. However components of the system are not organisationally-bounded to local government and include other public bodies (e.g. the NHS), third and private sector actors.

The boundaries of the system are as follows:

- Children and young people aged 0-25
- Children and young people living within the geographical footprint of a local authority
- All aspects of child health
- All dimensions of inequalities
- Determinants of child health outcomes that are amenable to change at the local level
- Outcomes, children, and inequalities:

The map is concerned with all aspects of child health (for example obesity, mental health, oral health, infectious diseases…) for children and young people (CYP) aged 0-25 years.

We also hope to reflect all aspects of inequality. Some aspects of inequality are on the map e.g. socio-economic status is represented by nodes such as ‘household income’ and ‘area-level deprivation’, as these are somewhat amenable to change through local intervention (through, for example, provision of welfare and benefits advice, improving employment and training opportunities, economic investment in deprived areas etc).

There are some population groups for whom their personal characteristics and circumstances are less amenable to change by local agencies (e.g. local governement, NHS or CCG), and will likely impact on health and wellbeing outcomes. For these groups, some factors on the map will have varying importance, and the map should be viewed through an ‘inequality lens’ according to these different population groups of children and young people. As an example, the factors on the map concerned with ‘safe places to play’ and ‘safe places to socialise’ will have varying importance for CYP of different ages, genders and (dis)ability; what ‘service accessibility’ means will differ for CYP from varying population groups.

We list below the personal characteristics and circumstances that participants in our two local areas, and in the last round of the survey, indicated should be included alongside the map. They include:

- Age
- Ethnicity
- Cultural and religious background
- Low birth weight
- Gender identity
- Sexual identity
- CYP from military families
- Young/teenage parents
- Young carers
- Care leavers
- CYP in care (including those placed out of the local authority area)
- CYP at risk
- CYP experiencing transition (of any type)

- CYP living with previously unsurvivable illness
- CYP with physical disability
- CYP with intellectual disability
- CYP with autism spectrum disorder
- CYP with special educational needs and disability (SEND)

This list is likely not exhaustive but is intended to encourage users to consider the differential effects of factors on the map on differing population groups. In the first round of the survey, respondents added:

- CYP with experience of ACEs
- CYP with complex and/or long term physical health conditions
- CYP with complex and/or long term mental health conditions (physical and mental have been split into separate factors)
- CYP with genetic predisposition to disorder(s)
- Asylum seeker/refugee status/citizenship status
- CYP who are members of Gypsy, Roma and Traveller communities

- Please tell us below if you think this list is helpful or not in encouraging users to interpret the system map according to the needs of differing population groups of CYP

- Is there another (better) approach to ensuring inequalities in health outcomes for such groups are considered in our system map?

Map domains and structure

The factors on the map identified through our work in two local authorities have been organised into six domains by adapting a framework from the literature (Goldfeld, S., Woolcock, G., Katz, I. et al. Neighbourhood Effects Influencing Early Childhood Development: Conceptual Model and Trial Measurement Methodologies from the Kids in Communities Study. Soc Indic Res (2015) 120:197). Factors within each domain are colour coded on the map.

Factors in the physical domain are those concerned with the physical environment, such as transport, housing, green space and local amenities and are intended to encourage users to consider how the physical environment can be designed or changed to support better child health outcomes.

Factors in the social domain are concerned with the people around CYP who influence health behaviours and norms, including parents and families, peer groups, and local communities. It also includes the influences of people CYP encounter online and in the media.

The economic domain includes factors at the household and local area level that influence the economic resources available to support CYP, including household income, local area deprivation, and local training and employment opportunities.

The services domain considers factors that influence the quality, quantity, accessibility and coordination of services across a range of areas. These include the services most obviously concerned with CYP and/or health (e.g. education, health, social care) but also other services which can have influence (e.g. transport; licencing; planning). It includes the public, private and third sectors.

These five domains have obvious interactions; governance at the local level will impact on the design and delivery of services; for example, local economics will determine the resources available to improve the physical environment. There are also interactions within factors in each domain. Factors in each domain are linked on the map if they directly affect each other. The map has links between factors within the same domain (and colour-coded to that domain) and across different domains (black links).

Respondents to our first survey have queried an absence of direct link between some factors. In some cases factors are linked in groups e.g. in the example below there is no direct link between ‘community health norms’ and ‘family health status’, but they are both linked via ‘family health norms'. [*example shown]*

It may be feasible to consider a direct link between these nodes (and others), but we are balancing readability with specificity: too many direct links make the map unreadable.

Please note that the size of the node does not indicate importance or significance.

Finally the personal domain is comprised of CYP health behaviours and other outcomes that are influenced by factors in these five previous domains.

Our map does not show causality, or direction of impact, between factors on the map. Several respondents to the first round of this survey queried this choice. There are two reasons why we have not attempted to show the direction of impact: a) complexity – the map is complex, with over 150 individual factors and adding causality would increase this and b) we do not always have the data to indicate causality. The map was developed through qualitative workshops and while participants indicated links they did not always indicate direction. We agree that further development of the map in any future iterations should consider causality and directional links.

Now we have explained how the map is structured, we would like to share the changes made since the last survey and ask for your views on this latest version.

[Each domain was then shown in turn. The service domain is shown as an example. The remaining five domains were shown in turn, with the exact same questions.]

The full services domain is shown below (we only show links between factors in this domain in the diagram below; many will also link to factors in other domains and you might want to refer to the full map (click here if you need to open it again).

Summary of changes in response to feedback from round 1:

Several respondents wanted to add more services to this domain, particularly specific health services. Please note we have deliberately limited the specificity of services shown, particularly those in health, which are summarised as primary, secondary, tertiary and public health services in order to make the map readable.

- We have added ‘health visiting’ as a service type.
- amended ‘residential/foster care’ to include ‘other placement types’
- removed a specific reference to ACEs in staff training and development
- added ‘children’s centres’

- added ‘library services’
- added ‘availability of affordable childcare’
- added ‘continuity between child and adult services’
- added ‘school policies and procedures’.

- Please tell us below if you agree or disagree with the changes made to this domain, and any other comments you may have on the structure of this domain.

Core determinants of child health inequalities outside of the immediate influence of agencies at the local level:

The factors on the map should be within influence and/or control of agencies at the local level (e.g. local government, NHS or CCG).

There are some factors identified by participants during our work with two local areas that impact on children and young people's health, but are not within the immediate influence or control of agencies at the local level. They include factors such as climate change, national government funding formulae, and the rural/urban geography of the local area.

During the first round of the survey, we referred to these as ‘edge factors’. However, feedback from survey respondents strongly indicated that some of these determining factors were so important to CYP health and wellbeing that the term ‘edge’ was misleading. However, it is important to have them included in any visual representation of the child health system at the local level to ensure that the wider context (and constraints) in which local agencies are operating is made clear.

We suggest re-naming ‘edge factors’ as the core determinants of child health inequalities outside of the immediate influence of agencies at the local level.

- Please tell us below if you agree or disagree with this change.

Factors listed in this category during the first round of the survey were

- Brexit
- Climate change
- Austerity
- National government policy and statutory guidance
- National government funding formulae
- Statutory inspection regime(s)
- Welfare reform
- Rural/urban geography of area

The following factors were added by respondents in the first round of the survey:

- National social/political/cultural environment
- Societal attitude to children and young people
- Innovation and technological advancement

- Please tell us below if you agree with these additions, and if there are further core determinants of child health inequalities outside of the immediate influence of agencies at the local level that you would like to see added.

We also asked respondents if these factors should be represented as separate nodes on the side of the map, but not linked to the map itself; as separate nodes on the side of the map, with links to nodes on the map; or not be on the map at all. The majority of respondents wanted them represented somewhere alongside the map but there were mixed views on whether they should be linked to factors on the map.

We suggest that these core determinants are listed at the side but not linked, as the number of links required would further increase the complexity of the map.

- Please tell us below if you agree or disagree with this approach to representing the core determinants of child health inequalities outside of the immediate influence of agencies at the local level:

Utilisation of the system map:

- Who do you anticipate might use this map (e.g. policy makers, practitioners or others)?
- Could you make use of this map in your own work? If yes, please tell us how.
- If using this map in a particular locality (such as a specific local authority), do you think that a local remapping process would be necessary? If yes, would your locality need assistance to remap or is there local expertise to facilitate this process?
- How useful might the current map be to support discourse with partners and the public around reducing child health inequalities?
- How useful might this map be to inform potential strategies for intervention or action?
- Do you have any other comments about the utility of the map? What works well or not so well?

Thank you:

Thank you, you have completed the survey.

The research team will collate the feedback from all respondents and make final amendments to the map based on these responses. We will be reporting on this study and making the final map publicly available later this year and will share this with you as soon as it is available.

If you have any questions about this study, we would love to hear from you. Please contact the research team by emailing tricia.jessiman@bristol.ac.uk
