# Supplementary material for: A systems map of the determinants of child health inequalities in England at the local level
Source: PLoS One. 2021 Feb 12;16(2):e0245577. doi: 10.1371/journal.pone.0245577 (PMC7880458; doi:10.1371/journal.pone.0245577)
Supplement: S2 Appendix — (PDF) [file pone.0245577.s002.pdf]

Mapping the child health system at a local level to reduce health inequalities

Personal characteristics and circumstances

For these groups of CYP, some factors on the map will vary in importance, and the map should be viewed through an 'inequality lens' according to these different populations of CYP

- Age
- Ethnicity
- Cultural and religious background
- Low birth weight
- Gender identity
- Sexual identity
- CYP from military families
- Young/teenage parents
- Young carers
- Care leavers
- CYP in care (including those placed out of the local authority area)
- CYP at risk
- Young offenders
- Asylum seeker/refugee status/citizenship status
- CYP who are members of Gypsy, Roma and Traveller communities
- CYP experiencing transition (of any type)
- CYP living with previously unsurvivable illness
- CYP with physical disability
- CYP with intellectual disability
- CYP with autism spectrum disorder
- CYP with special educational needs and disability (SEND)
- CYP with experience of ACEs
- CYP with complex and/or long term physical health conditions
- CYP with complex and/or long term mental health conditions
- CYP with genetic predisposition to disorder(s)

Domain key: Physical Domain | Governance Domain | Economic Domain | Social Domain | Services Domain | Personal Domain

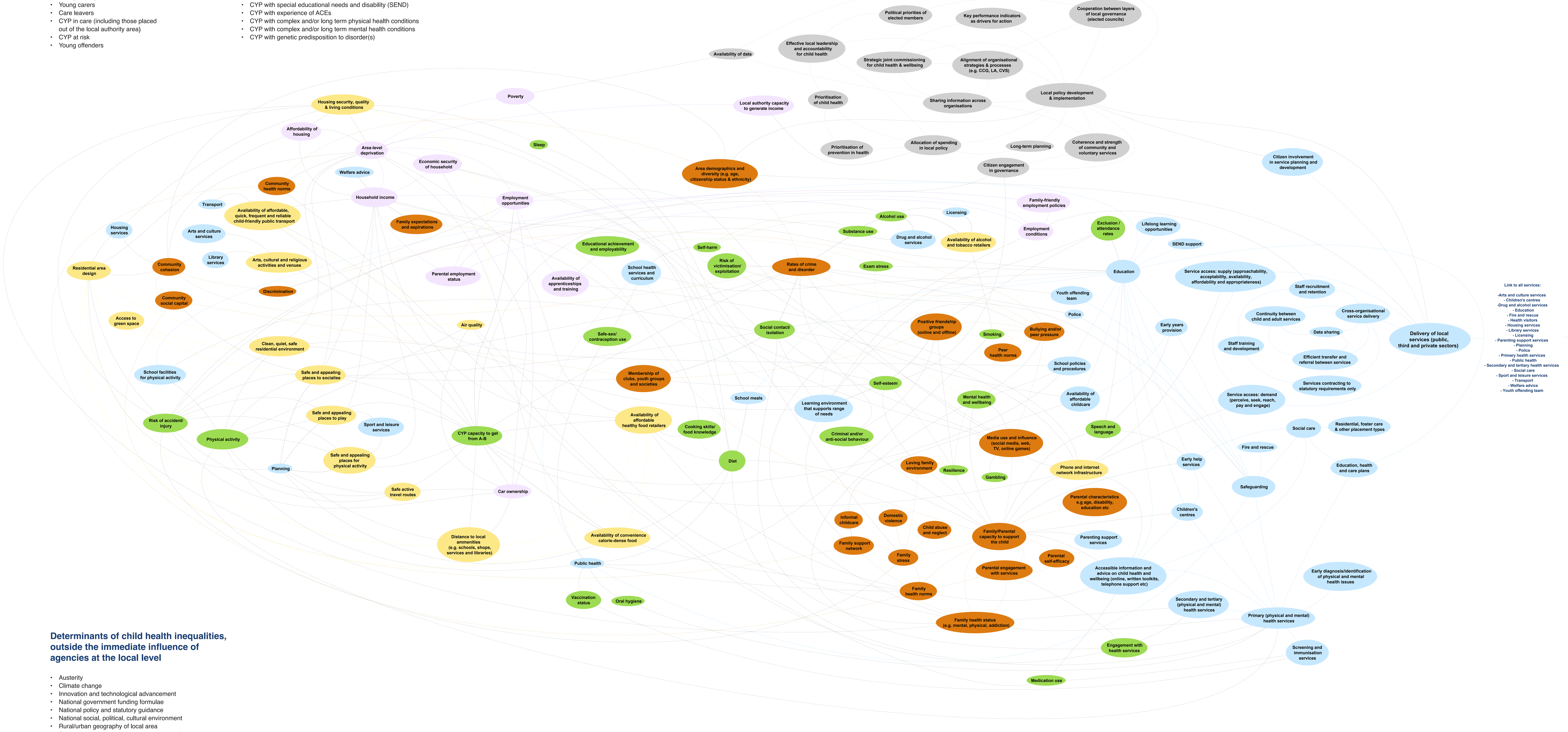

Determinants of child health inequalities, outside the immediate influence of agencies at the local level

- Austerity
- Climate change
- Innovation and technological advancement
- National government funding formulae
- National policy and statutory guidance
- National social, political, cultural environment
- Rural/urban geography of local area
- Societal attitude to children and young people
- Statutory constraints
- Statutory inspection regime(s)
- Welfare reform
